# The Impact of Virtual Reality Training on the Quality of Real Antromastoidectomy Performance

**DOI:** 10.3390/jcm9103197

**Published:** 2020-10-02

**Authors:** Wojciech Gawęcki, Magdalena Węgrzyniak, Patrycja Mickiewicz, Maria Bratumiła Gawłowska, Marcin Talar, Małgorzata Wierzbicka

**Affiliations:** 1 Department of Otolaryngology and Laryngological Oncology, Poznań University of Medical Sciences, 60-355 Poznań, Poland; m.wegrzyniak89@gmail.com (M.W.); otosk2@gmail.com (M.W.); 2 WSB University, 41-300 Dąbrowa Górnicza, Poland; p.mickiewicz86@wp.pl; 3 Medicus sp. z o.o., 50-224 Wrocław, Poland; mgawlowska@medicus.com.pl (M.B.G.); mtalar@medicus.com.pl (M.T.); 4 Poznań University of Medical Sciences, 61-701 Poznań, Poland; 5 Faculty of Health Sciences, Pomeranian Medical University in Szczecin, 71-210 Szczecin, Poland

**Keywords:** virtual reality, temporal bone surgery, antromastoidectomy, active assisting, supervised surgery

## Abstract

Background: The aim of this paper is to analyze the results of virtual reality (VR) antromastoidectomy simulation training and the transferability of the obtained skills to real temporal bone surgery. Methods: The study was conducted prospectively on a group of 10 physicians, and was composed of five VR simulation training sessions followed by live temporal bone surgery. The quality of performance was evaluated with a Task-Based Checklist (TBC) prepared by John Hopkins Hospital. Additionally, during every VR session, the number and type of mistakes (complications) were noted. Results: The quality of performance measured by the TBC increased significantly during consecutive VR sessions. The mean scores for the first and fifth sessions were 1.84 and 4.27, respectively (*p <* 0.001). Furthermore, the number of mistakes in consecutive VR sessions was gradually reduced from 11 to 0. During supervised surgery, all the participants were able to perform at least part of an antromastoidectomy, and the mean TBC score was 3.57. There was a significant strong positive correlation between the individual results of the fifth VR session and the individual results of supervised surgery in the operating room (r_p_ = 0.89, *p =* 0.001). Conclusions: Virtual reality for temporal bone training makes it possible to acquire surgical skills in a safe environment before performing supervised surgery. Furthermore, the individual final score of virtual antromastoidectomy training allows a prediction of the quality of performance in real surgery.

## 1. Introduction

To date, temporal bone training has been largely based on traditional dissection of cadaveric temporal bones through in-house training or participation in national or international temporal bone courses [1]. Training facilities are in the vast majority of European ENT Departments dealing with otosurgery, but due to the poor availability of human temporal bones, access for trainees is limited [1,2,3]. Virtual reality (VR) simulation and artificial temporal bone models have been gaining popularity as training supplements to “wet” dissection in many institutions, and nowadays, multiple VR simulators are commercially available [4,5,6,7,8].

VR training has many important advantages. It provides a wide range of anatomic variants and enables failures without consequences and unlimited practice [9,10,11,12,13]. The key components of the training are to avoid exceeding the anatomical boundaries of a complete mastoidectomy and to avoid violating vital structures [6]. Simulation-based technical skills training must present a good balance between automatic scoring, drilling time and self-assessment [14]. A simulator-integrated tutor function has significantly improved performance in these areas [6], and the number of objective assessment tools for self-directed practice has been reported [15]. A feasible alternative is final-product analysis or a simulator-based automated assessment and feedback scoring system [14,15]. For a combined metrics-based score (MBS), a significant discriminative ability between experienced surgeons and residents was demonstrated; nevertheless, it failed to measure or encourage safe routines [14]. Moreover, at present, the automated feedback based on metrics in VR simulation does not have a sufficient empirical basis and has not been generally accepted for use in training or certification [16]. Thus, the VR simulation training requires supplemental approaches, feedback, improved self-assessment tools and raising awareness of real operating field challenges. Nearly all previous VR studies were limited because that they did not assess the effect of simulation training on “real life” performance in an operating room (OR). 

Thus, the aim of this paper is to analyze the results of VR simulation training for antromastoidectomy, which is one of the basic otosurgical procedures [17], and to evaluate the transferability of the obtained skills to the real temporal bone operating field, measured by a specialist’s assessment of a supervised surgery.

## 2. Experimental Section

### 2.1. Study Design and Setting

**Figure 1:**
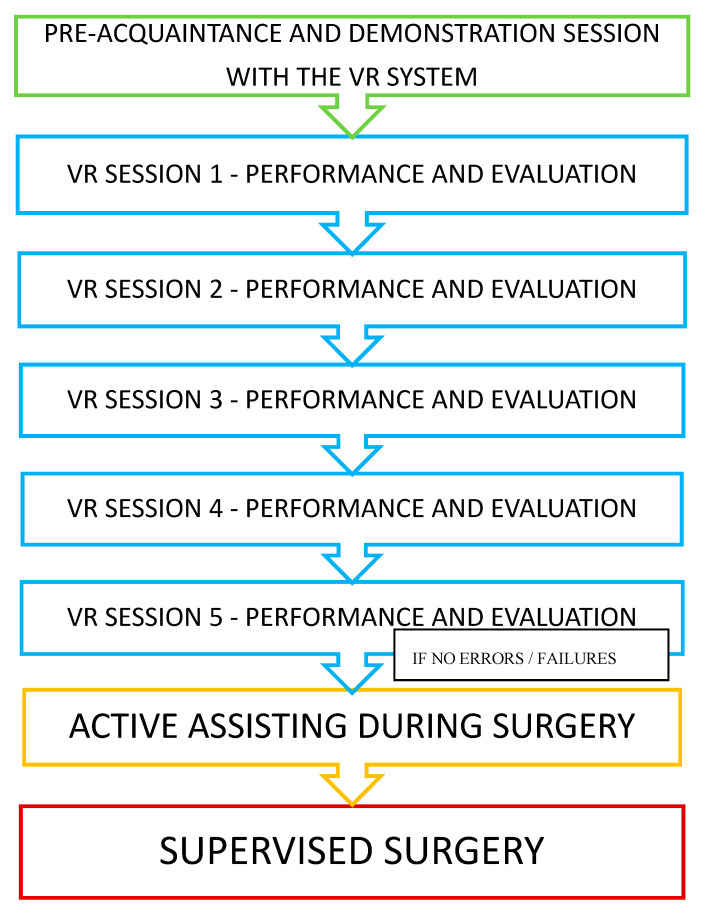
Study design.

The study was conducted prospectively, with five VR-simulation training sessions (separated by 4–5-week breaks), arranged before the real temporal bone surgery in the OR (Figure 1). Participants performed a series of virtual dissections after the preacquaintance and demonstration session with the VR system. During every VR session, the participants had to perform a virtual antromastoidectomy (cortical mastoidectomy). After the completed VR training, their knowledge was checked while actively assisting in a mastoid process surgery. The participants’ performance was then scored during a supervised surgery—an antromastoidectomy performed as a part of a cochlear implantation. Participants were allowed to assist or perform any real surgical procedures only if they were able to perform the fifth VR simulation without any mistakes (complications). The study was performed between October 2018 and August 2019, and the regional ethics committee deemed this study to be exempt.

### 2.2. Materials

The participants consisted of 10 physicians: four ENT specialists experienced in rhinology or head and neck oncology, and six otorhinolaryngology residents. None of the participants were experienced in otosurgery. They had never performed an antromastoidectomy before, neither in a VR simulator or cadaver specimen nor in a real patient in an OR. All of them had assisted in many (more than 30) otological procedures (tympanoplasties, cholesteatoma surgeries and cochlear implantations). Two experienced otosurgeons (with more than 10 years of experience and more than 1000 performed middle-ear surgeries) participated in the study as supervisors for all the participants.

### 2.3. VR Simulator

**Figure 2:**
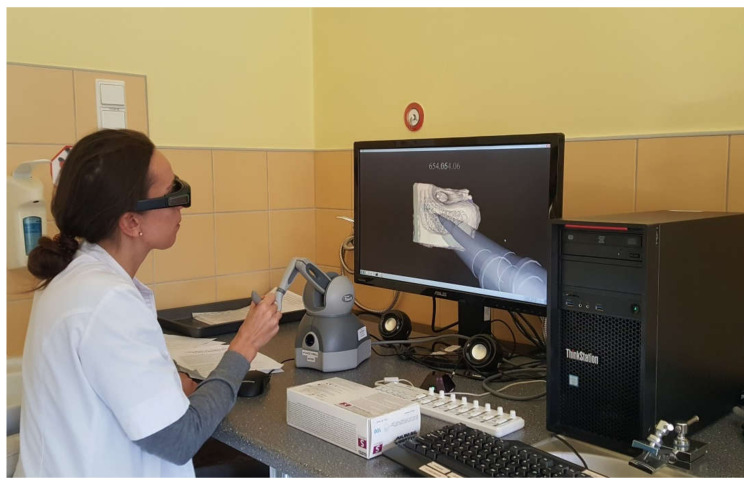
Virtual reality (VR) temporal bone surgery.

The participants were presented with a graphical representation of the bone via a temporal bone surgery simulator composed of a Geomagic Touch Haptic Device from 3D Systems, a MIDI controller from KORG, NVidia 3D Active glasses, a monitor with 3D technology, workstation PC and software created by a team from The University of Melbourne. The system was validated for its user interface and content [7,18,19]. The models were generated from microCT human temporal bone scans with a voxel resolution of 96 × 96 × 96 µm [20]. Thanks to the 3D Active glasses, 3D monitor and dedicated software, the system gives access to a three-dimensional view. In this system, the drill handpiece with a cutting or polishing tip is visible on the screen. The tips can be changed depending on the needs. The drill handpiece and the tip accurately reflect what the surgeon can see in the real operating field. The handle of the haptic device resembles the weight of a drill used in real surgical operations. While working with the haptic device, the user feels vibrations while drilling, resistance to stronger pressure on the hard bone structures in the 3D model and additionally, hears the changing sound. The remains of the drilled bone are invisible to the user. The model can be rotated in any direction. In our study, we selected one simple (and always the same) model of a temporal bone with very typical anatomical features, which reflects most of the population’s temporal bones. A study participant during a VR session is presented in Figure 2.

### 2.4. Evaluation of VR Sessions

The quality of performance was evaluated by a Task-Based Checklist (TBC) prepared by John Hopkins Hospital and described by Francis et al. [21]. The TBC is used to evaluate the performance of a series of individual surgical steps on a 5-point Likert scale—from 1 (unable to perform) to 5 (performs easily with good flow). The whole checklist, prepared for evaluation of middle-ear surgery, is composed of 22 items, grouped into seven major tasks. In our study, 11 items, grouped into the following four tasks concerning cortical mastoidectomy were evaluated: (1) initial bone cuts, (2) defining anatomic limits, (3) open antrum and (4) thin posterior EAC (external auditory canal) cortex. Additionally, during every VR session, the number and type of mistakes (complications) were noted. Every simulation was assessed by both supervisors, and a mean of two ratings was calculated. The time of the session was limited to 40 min.

### 2.5. Active Assisting During Surgery

The participants who had no failures in the fifth VR simulation were allowed to actively assist during a surgery. The surgery was always performed by the same expert supervising the study (WG). During active assisting surgery, the participants were assessed on their task of verbally listing the following surgical steps in the proper order: (1) removal of the cortical bone, (2) opening of the mastoid air cells, (3) visualization of the dura and sigmoid sinus, (4) finding the antrum, (5) finding the lateral semicircular canal and (6) finding the short process of the incus. Furthermore, they were asked to give the proper names of the following anatomical details indicated by the otosurgeon: (1) the posterior wall of the external auditory canal, (2) the middle cranial fossa dura, (3) the sigmoid sinus, (4) the lateral semicircular canal, (5) the incus and (6) the course of the facial nerve.

### 2.6. Supervised Surgery

Supervised surgery was always performed under the guidance of the same supervising otosurgeon (WG), and was always evaluated by both supervising experts. Participants had to perform an antromastoidectomy as part of a cochlear implantation procedure. The procedures were performed only on patients with normal anatomy of the temporal bone, as confirmed by a preoperative CT. The quality of performance was evaluated by the TBC in the same way as the VR training sessions. During real surgery, the safety of the patient was crucial and no mistakes were allowed, thus the surgery would be stopped (not completed by the participant) if a supervisor decided it was potentially dangerous or the participant did not feel confident enough to continue. The time limit was 40 min. 

### 2.7. Statistical Analysis

Statistical analysis was carried out in R software, version 3.5.1, 2018 (http://cran.r-project.org), The R Foundation for Statistical Computing Institute for Statistics and Mathematics, Vienna, Austria. Normality of TBC data was confirmed based on Shapiro–Wilk test results, as well as based on skewness and kurtosis values and visual assessment of histograms. TBC results between sessions were compared with MANOVA, a Mauchly test was used to check data sphericity, and Greenhouse–Geisser and Huynh–Feldt corrections for departure from sphericity were applied. To identify specific sessions that had a significantly different TBC result, a post-hoc test was used (a paired t-test with Bonferroni correction for multiple comparisons). Correlation between the last VR session and the OR session for TBC was analyzed with Pearson’s correlation coefficient (r_p_). The intraclass correlation coefficient (ICC) was computed to assess the agreement between the two raters. All tests were based on α = 0.05.

## 3. Results

The group of 10 participants managed to complete the course of five VR sessions, and all were allowed to take part in active assisting during a surgery, and further, in a supervised surgery.

### 3.1. The Quality of the Performance of VR Simulation

**Figure 3:**
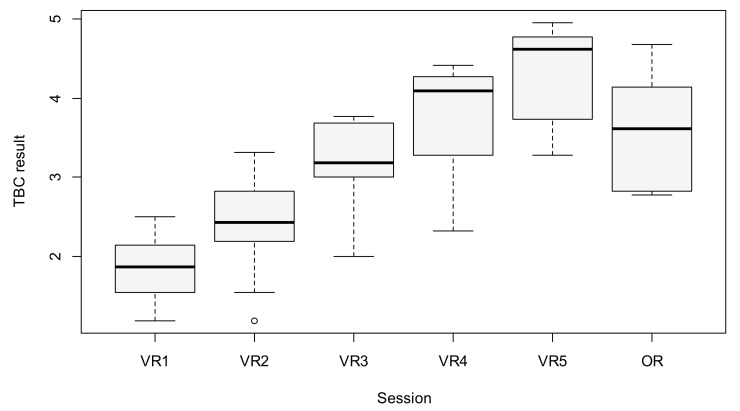
Boxplot of TBC results between sessions (the circle above VR2 represents an outlier observation).

The quality of performance measured by TBC increased significantly during consecutive VR sessions. The mean scores for the first and fifth sessions were 1.84 and 4.27, respectively (*p <* 0.001). There was a significant difference between all five VR sessions in the average TBC result, as based on the MANOVA analysis (*p <* 0.001). To identify which sessions were responsible for this difference, a post-hoc test was conducted. The results are presented in Table 1 and Table 2, and in Figure 3. 

### 3.2. The Number of Mistakes in VR Simulations 

**Figure 4:**
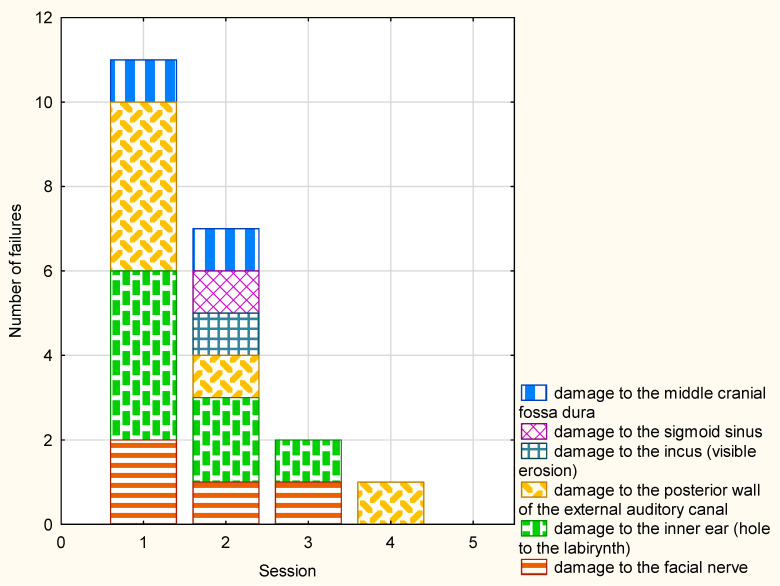
The number and characteristics of the failures during VR sessions.

In consecutive VR sessions, the number of mistakes was evidently reduced. Altogether, 11 mistakes were observed in the participants’ first session (among them, two injuries of the facial nerve and four injuries of the inner ear). No mistakes were observed in their fifth session. The number and characteristics of the failures are presented in Figure 4.

### 3.3. Evaluation of Active Assisting During Surgery

Due to the fulfillment of the threshold criterion of no mistake during the final VR session, all the participants were allowed to take part in active assisting during a surgery. During it, all the participants were able to list the surgical steps in the proper order, and all but one were able to give the proper names of all the indicated anatomical details. Only one resident had a problem giving the proper name of the incus, but named the other structures properly.

### 3.4. Evaluation of a Supervised Antromastoidectomy Performance

All of the participants of the study were then allowed to perform a supervised surgery. The mean score was 3.57, which was better than for the first, second and third VR sessions (the difference for Sessions 1 and 2 was statistically significant), although worse than the fourth and fifth VR sessions (for Session 5, the difference was statistically significant). The results are presented in Table 1 and Table 2, and in Figure 3. Five of the ten supervised surgeries were not completed by the participant, requiring the supervisor to finish. Reasons included: (1) participant’s decision (lack of self-confidence to continue) in two cases, (2) supervisor’s decision (surgery being potentially dangerous) in one case and (3) exceeding the time limit in two cases. 

### 3.5. Correlation of the VR Final Score and Real Antromastoidectomy Performance

**Figure 5:**
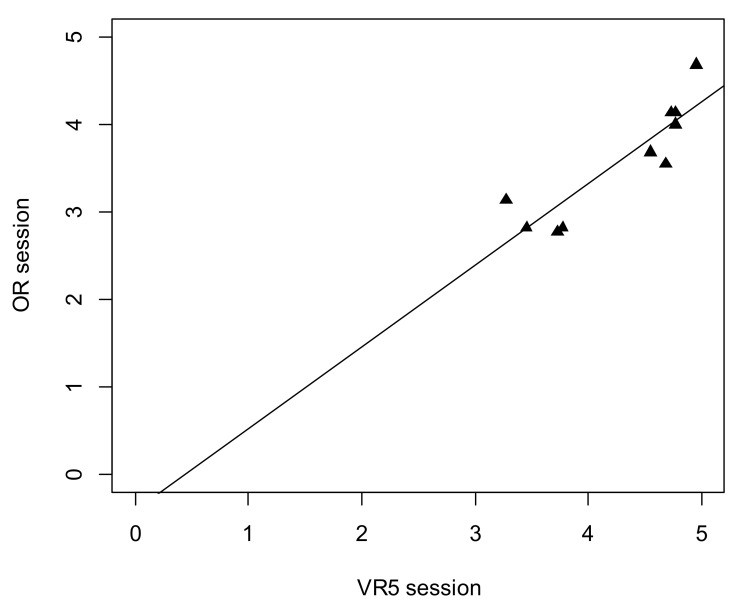
Scatterplot for TBC result between VR5 session and operating room (OR) surgery.

There was a significant strong positive correlation between the individual results of the fifth VR session and the individual results of supervised surgery in the OR (r_p_ = 0.89, *p* = 0.001). The details are presented in Figure 5.

### 3.6. Inter-Rater Agreement

There was significant excellent agreement between raters for all the sessions, using the two-way random effect models, ICC = 0.995; CI_95_ [0.981; 0.998]; *p <* 0.001.

## 4. Discussion

The aim of this paper was to analyze the results of VR simulation training with special regard to the transferability of the obtained surgical skills to the real temporal bone operating field. The influence of VR training on different real surgeries, i.e., colonoscopy, laparoscopic camera navigation and endoscopic sinus surgery, showed that the participants who reached proficiency in simulation-based training performed better in a patient-based setting than their counterparts who did not have such training, and furthermore, simulation-based training was equally as effective as patient-based training [22]. In temporal bone surgery, human cadaveric dissection has been the gold standard of training for a long time because it closely mimics real-life surgical conditions [23]. However, contemporary otologic training is primarily acquired in an OR because the instruction and practice of using a cadaveric temporal bone is less consistently available to trainees today [11]. Furthermore, artificial bone models, which are popular in multiple medical disciplines (dental implants, maxillo-facial surgery, orthopedics), are not widely used in otosurgery because of some deficiencies in comparison to real temporal bone. However, they can be an alternative for beginners [24,25].

Fortunately, a new option—VR temporal bone training—has been gaining importance for the past few years, and nowadays, VR is a well-established and useful adjunct to traditional cadaveric dissection of temporal bone for trainees [6,19,26]. Such training may play an important and increasing role in education in the future, but under certain conditions and according to some standards [14]. This way of training is reportedly used in many leading training departments in Europe, and most of the remaining departments expect to implement VR simulation for temporal bone training into their residency programs in the near future [1].

The question arises whether this kind of training would allow participants to skip ahead, from VR to gradually more advanced assists and supervised surgeries, and consequently to replacing cadaveric temporal bone dissections completely or significantly. The benefits of VR training in antromastoidectomy performance were shown in cadaveric temporal bone dissection [19]. Andersen et al. proved that even two hours of self-directed VR simulation training were effective in increasing cadaveric dissection mastoidectomy performance. The conclusion is that mastoidectomy skills are transferable from VR simulation to traditional dissection [6]. The impact of VR training on real middle-ear surgery performance was checked by Al-Noury, who found that in previously simulated cases, the residents scored higher, were faster and more confident and required fewer instructions [26].

However, the impact of VR training on real surgery has not been sufficiently proved. Thus, in this paper, we aimed at answering whether VR is an effective training instrument that enables physicians to obtain skills sufficient for surgery in a real temporal bone operating field. For this purpose, we planned a training program of five sessions separated by 4–5-week breaks. This was based on the observations described by Andersen et al., who found that the mastoidectomy skills acquired under time-distributed practice conditions were retained better than skills acquired under massed practice conditions [27]. 

The tool we used was a validated Task-Based Checklist (TBC), prepared by John Hopkins Hospital [21]. According to Sethia et al., who reviewed the literature for assessment of performance for mastoidectomy, this tool possesses the most validity evidence of those reviewed [15]. However, as TBC does not take into account the number and type of performed mistakes (intraoperative complications) during a surgery, we added such analysis in every VR session. We found that the VR lab is perfect to facilitate repeated training in mastoid basic surgery. During consecutive sessions, the quality of performance increased, and most importantly, the number of mistakes (intraoperative complications) gradually decreased to zero. This effect can be explained by both an improvement in anatomical knowledge and surgical skills after repeated training. Active assisting during surgery confirmed excellent anatomical and surgical knowledge obtained during the VR antromastoidectomy course. All of the participants were then allowed to perform a supervised surgery, and all of them managed to perform at least part of an antromastoidectomy, with a mean score that compared to that between their third and fourth VR sessions. Such results indicate the importance of VR training (results significantly better than in the first and second VR sessions), and also the influence of the change of working environment from VR to real surgery (where results were significantly worse in comparison to the fifth, and last, VR session), which is fully understandable. As safety during the surgery and the wellbeing of the patient are always the most important aspect, it is understandable that either the participant or supervisor decided to stop when the safety of the surrounding structures was potentially endangered or if the surgery time was exceeded. No complications occurred during the surgeries performed by the participants or in combination with the supervisors. We have shown that just five sessions of VR temporal bone training might be enough to enable physicians to obtain the skills sufficient to perform a supervised antromastoidectomy. Furthermore, we found a significant strong positive correlation between the individual results of the fifth VR session and the individual results of the supervised surgery in the OR, which shows that the individual final score of VR training allows a prediction of the quality of performance in a real surgery.

This study has several limitations. First, the VR simulator used in the study was relatively simple. We are aware that making VR simulations more realistic could improve the quality of surgery in the operating room. Second, the lack of training on temporal bones from cadavers after the last VR session and immediate switch to a real operation is a downside of this study, but at the same time, we are trying to prove the possibility of bypassing that intermediate training stage, as it is increasingly difficult to organize. The most serious study limitation is its small sample size and single-center design. 

This study also has several advantages. The most noteworthy is the repeated use of the same VR model and comparing the learning curve and gained skills with the in vivo operating field. 

To summarize, VR temporal bone training can address needs in continuing education and competency-based residency training, but there is still the open question of whether it ultimately becomes a component of the certification process. We believe that in the future, this form of repeated VR simulation can even totally replace the cadaveric temporal bone lab and allow residents to proceed to surgery under specialist guidance, preselected by active assisting during a surgery. Systematic integration of training using VR simulation is possibly the future direction of surgical first steps.

## 5. Conclusions

Virtual reality for temporal bone training makes it possible to acquire surgical skills in a safe environment before performing supervised surgery. Furthermore, the individual final score of virtual antromastoidectomy training allows a prediction of the quality of performance in a real surgery.

## Figures and Tables

**Table 1:** Task-Based Checklist (TBC) results by session (VR–virtual reality surgery; OR–supervised surgery in operating room; Q1–lower quartile; Q3–upper quartile; *n* = 10).

Session	Mean	SD	Median	Q1	Q3	Min.	Max.
VR1	1.84	0.41	1.86	1.56	2.08	1.18	2.50
VR2	2.41	0.68	2.43	2.20	2.80	1.18	3.32
VR3	3.10	0.64	3.18	3.02	3.60	2.00	3.77
VR4	3.70	0.76	4.09	3.31	4.23	2.32	4.41
VR5	4.27	0.64	4.61	3.74	4.76	3.27	4.95
OR	3.57	0.67	3.61	2.90	4.10	2.77	4.68

**Table 2:** TBC change between sessions (MANOVA post-hoc test; MD—mean difference in TBC between two sessions calculated as the later session mean minus the previous session mean, with a 95% confidence interval (CI); *p*–individual paired t-test *p*-value; *p_adj_*–paired t-test *p*-value adjusted for multiple comparisons (Bonferroni correction)).

Sessions Comparison	MD	95% CI	*p*	*p_adj_*
VR1–VR2	0.57	(0.32; 0.82)	0.001	0.009
VR1–VR3	1.26	(0.96; 1.56)	<0.001	<0.001
VR1–VR4	1.85	(1.47; 2.24)	<0.001	<0.001
VR1–VR5	2.43	(2.07; 2.78)	<0.001	<0.001
VR1–OR	1.73	(1.27; 2.19)	<0.001	<0.001
VR2–VR3	0.69	(0.53; 0.84)	<0.001	<0.001
VR2–VR4	1.28	(0.99; 1.57)	<0.001	<0.001
VR2–VR5	1.85	(1.49; 2.22)	<0.001	<0.001
VR2–OR	1.16	(0.64; 1.68)	0.001	0.010
VR3–VR4	0.60	(0.40; 0.79)	<0.001	0.001
VR3–VR5	1.17	(0.90; 1.44)	<0.001	<0.001
VR3–OR	0.47	(0.04; 0.91)	0.036	0.534
VR4–VR5	0.57	(0.40; 0.74)	<0.001	<0.001
VR4–OR	−0.12	(−0.49; −0.24)	0.467	>0.999
VR5–OR	−0.70	(−0.92;−0.47)	<0.001	0.001
